# Clinical and epidemiological characteristics of adult hand, foot, and mouth disease in northern Zhejiang, China, May 2008 – November 2013

**DOI:** 10.1186/1471-2334-14-251

**Published:** 2014-05-10

**Authors:** Xin-guang Yin, Hui-xing Yi, Jin Shu, Xing-ju Wang, Xiao-jun Wu, Ling-hua Yu

**Affiliations:** 1 Centre for Gastroenterology and Hepatology, The First Affiliated Hospital of Jiaxing College, Jiaxing 314001, Zhejiang Province, People’s Republic of China; 2 Intensive Care Unit, The Second Affiliated Hospital of Zhejiang University, Hangzhou 31009, Zhejiang Province, People’s Republic of China; 3 Intensive Care Unit, The First Affiliated Hospital of Jiaxing College, Jiaxing 314001, Zhejiang Province, People’s Republic China; 4 Department of Infectious Diseases, Centre for Gastroenterology and Hepatology, The First Affiliated Hospital of Jiaxing College, 1882 Central-South Road, Jiaxing 314001, Zhejiang Province, People’s Republic China

**Keywords:** HFMD, Adult, Enterovirus, EV71, CA16, Genotype

## Abstract

**Background:**

Hand, foot, and mouth disease (HFMD) is an infectious disease typically caused by enterovirus 71 (EV71) and Coxsackievirus A16. The incidence of HFMD appears to be increasing across the Asia Pacific region, with deaths occurring predominantly among children. Therefore, most HFMD reports focus on children and few have studied HFMD in adults. However, more adult HFMD cases may be seen in the foreseeable future as a result of global warming, continued viral evolution, and an increase in traveling. Thus, this study investigated the clinical and epidemiological characteristics of adult HFMD.

**Methods:**

Case data of 49 adult HFMD patients who attended The First Affiliated Hospital of Jiaxing College, China from May 2008 to November 2013 were obtained. Socio-demographic data were collected through follow-up phone calls. Throat swab specimens were tested for enterovirus by quantitative reverse transcription-polymerase chain reaction and further confirmed by virus isolation assay. For 10 patients infected with EV71, the gene encoding the EV71 VP1 protein was sequenced and analyzed. Data from 8,354 child HFMD patients and 49 adult patients in the fever clinic of The First Affiliated Hospital of Jiaxing College during the same period were collected for comparison.

**Results:**

This study revealed that close contact with HFMD patients and poor personal hygiene consciousness were risk factors for adult HFMD. This study also found that EV71 subgenotype C4a was the most common pathogen associated with adult HFMD in this area. Furthermore, this study demonstrated several unique epidemiological characteristics of adult HFMD compared to child HFMD, such as the geographic and gender distribution of adult HFMD patients and HFMD seasonality.

**Conclusions:**

The findings in this study showed the potential threat of adult HFMD.

## Background

Hand, foot, and mouth disease (HFMD) is an acute infectious disease occurring mainly in children, and is characterized by fever and herpetic lesions on the hands, feet, or oral mucosa. Infants and children under 5 years of age are generally susceptible to HFMD, but the disease is rare in adults.

HFMD is caused by human enteroviruses, members of the Picornavirus family, which are transmitted through the fecal-oral route or via respiratory droplets [1,2]. Enterovirus 71 (EV71) and Coxsackievirus A16 (CA16) are the two major causative agents of HFMD. EV71 is a neurotropic virus that can cause severe neurological and cardiovascular complications in infected patients and can have a high fatality rate, which makes it a notable member of the enteroviruses [3,4]. CA16 is the other common pathogen that causes HFMD, and may be associated with myocarditis, pericarditis, and other severe diseases [5].

Adult HFMD is uncommon, and to date only one adult death has been reported [6]. However, a rising incidence of adult HFMD might be expected in the foreseeable future as the result of climate change, continued viral evolution, and an increase in global travel. Therefore, the purpose of this study was to investigate the clinical and epidemiological features of adult HFMD.

## Methods

### Patients and throat swab specimens

HFMD case data were obtained from The First Affiliated Hospital of Jiaxing College, which is the largest hospital in Northern Zhejiang Province, China. Sociodemographic data of adult HFMD patients were collected through follow-up phone calls. All individual-level data were anonymized. This study was approved by the Ethical Committee of Jiaxing College. Written informed consent for publication was obtained from each patient who provided clinical specimens or images.

A total of 49 adult (defined as 18 years old or older) HFMD cases from May 2008 to November 2013 were identified. Data of 49 adult patients who attended the fever clinic of The First Affiliated Hospital of Jiaxing College (but without HFMD) and 8,354 child HFMD patients during the same period were collected for comparison.

Throat swab specimens from 32 adult HFMD patients and 300 child HFMD patients from May 2008 to November 2013 were collected by trained medical personnel. Samples were preserved at -80°C.

### Clinical criteria

Clinical criteria for the diagnosis of HFMD cases were published by the Chinese Ministry of Health in 2008 [7]. Patients with the following symptoms were defined as having HFMD: fever, papules and herpetic lesions on the hands, feet, or oral mucosa; rashes on the buttocks or knees, inflammatory flushing around the rashes, and blisters with little fluid.

### Quantitative reverse transcription-polymerase chain reaction (RT-PCR)

All throat swab samples were tested using a fluorescence quantitative RT-PCR assay for EV71, CA16, and pan-enteroviruses. Viral RNA was extracted from samples using the QIAmpH Viral RNA Kit (Qiagen, Hilden, Germany) according to the manufacturer’s protocol. Complementary DNA was generated from the extracted RNA by reverse transcription with oligo (dT) primers (Life Technologies, Carlsbad, California, USA) and preserved at -20°C. Thermocycling conditions included an initial step of 2 min at 94°C, followed by 40 cycles of denaturing at 94°C for 30s, annealing at 60°C for 45 s, and elongation at 72°C for 1 min [8]. The primer pairs and probes used for amplification are listed in Table 1.

**Table 1:** Primer sequences

**Gene**	**Primer/Probe**	**Sequence**
Enterovirus	Forward	5′-GTGTCGTAACGGGTAACTCTGCA-3′
	Reverse	5′-CAATTGTCACCATAAGCAGCCA-3′
	Probe	5′-TAGAACCTACTACTTACTGTGTCCT-3′
EV71	Forward	5′-CCACAAGCCAGCGGGTAGT-3′
	Reverse	5′-AAACACGGACACCCAAAGTAGTC-3′
	Probe	5′-AACTCTGCAGCGGAAC-3′
CVA16	Forward	5′-ATCTGTATCGATCTGGGTTTTGC-3′
	Reverse	5′-AGTAAAGCGCCTTGGTGGAA-3′
	Probe	5′-ACGTTCAGTGTAACGCAA-3′
EV71-VP1	Forward	5′-GGTGCGCCCAACACAGCTT-3′
	Reverse	5′-CCGCCGCAATCACCAGGTT-3′

To confirm the RT-PCR results, some of the samples (six samples that were EV71 positive and one sample that was CA16 positive) were further cultured in human rhabdomyosarcoma cells (RD cells, ATCC HTB-139) and examined for the presence of infectious viruses.

### Sequence and phylogenetic analysis

Samples that were positive for EV71 were selected for genotyping. The gene encoding the VP1 protein was obtained by RT-PCR, and the primer pair for the EV71 VP1 gene is listed in Table 1. Amplification was performed in 35 cycles consisting of denaturing at 94°C for 1 min, annealing at 54°C for 30s, and elongating at 68°C for 2 min. PCR products were purified using the TaKaRa Agarose Gel DNA Purification Kit (TaKaRa, Dalian, China) and sequenced using an ABI Prism 3730 DNA Analyzer (Applied Biosystems, Carlsbad, California, USA).

Sequence alignment of the strains was performed with ClustalW analysis in Mega 5.2.2 (Molecular Evolutionary Genetics Analysis software, Tamura K, Peterson D, Peterson N, Stecher G, Nei M, and Kumar S 2011). A phylogenetic tree was constructed using the neighbor joining method with the Kimura two-parameter model of nucleotide substitution, and bootstrap analyses were performed on 1,000 replicates.

### Statistical analysis

Data were analyzed using the Chi-square test or Fisher’s exact test, and p < 0.05 was considered significant. Single-factor analysis and logistic regression analysis (likelihood ratio test) were used to explore risk factors for adult HFMD. All statistical analyses were performed using the statistical package R for OSX 2.15.2 (R Foundation for Statistical Computing, Vienna, Austria).

## Results

### General information of adult HFMD patients

During the period of this study, 49 patients met the criteria for adult HFMD. Epidemiological and clinical features are summarized in Table 2. The median age of patients was 27.5 years, 59.2% were female, 71.4% had children under 5 years of age, and 38.8% had recently had children diagnosed with HFMD. A total of 32.7% were living with more than four people in the home, and 22.4% had a living space less than 10 square meters per person.

**Table 2:** Risk factors for adult HFMD (single-factor analysis)

**Epidemiological characters**	**Adult HFMD patients**	**Adult fever clinic patients**	**χ**^**2**^	**P value**	**OR (95% CI)**
Gender			0.37	0.54	-
Male	20	23			
Female	29	26			
Residence			0.40	0.53	-
Urban	33	30			
Rural	16	19			
Occupation			-	0.51	-
Medical staff	5	4			
Civil servant	6	5			
Teacher	8	5			
Student	2	7			
Farmer	6	10			
Labor worker	14	13			
House-hold	8	5			
Education			0.38	0.54	-
High school or below	31	28			
College	18	21			
Family size			7.33	<0.01	4.27 (1.42 ~ 12.83)
≥4 persons	16	5			
<4 persons	33	44			
per capita living space			3.86	0.05	3.26 (0.96 ~ 11.07)
<10 square meter/person	11	4			
≥10 square meter/person	38	45			
Having separate toilet at home			0.71	0.40	-
Yes	45	47			
No	4	2			
Having children under 5-years-old			30.21	<0.01	12.81 (4.81 ~ 34.10)
Yes	35	8			
No	14	41			
Having children diagnosed as HFMD			20.35	<0.01	30.4 (3.87 ~ 238.99)
Yes	19	1			
No	30	48			
Keep pets			-	0.29	-
Cat	1	2			
Dog	5	11			
Others	1	1			
No	42	35			
Play mobile games			0.55	0.46	-
Yes	37	40			
No	12	9			
Sharing mobile with others			8.61	<0.01	6.13 (1.63 ~ 23.01)
Yes	14	3			
No	35	46			
Play mobile more than 1 hour per day			1.34	0.25	-
Yes	10	15			
No	39	34			

### Clinical characterization and laboratory diagnosis of adult HFMD cases

All 49 patients presented with herpetic lesions on the hands, feet, or oral mucosa, 16 (32.7%) had fever, and none had severe complications. No severe cases were found in this study, and the prognosis of adult HFMD was generally good. Four (8.2%) patients had a white blood cell count higher than 10,000/μL, and none had X-ray-confirmed pneumonia.

Throat swab specimens from 32 adult HFMD patients and 300 child HFMD patients were examined for human enteroviruses by quantitative RT-PCR assays. Of the 32 specimens collected from adult HFMD patients, 10 (31.3%) were positive for EV71, four (12.5%) were positive for CA16, and two (6.3%) were positive for other enteroviruses. Of the 300 child HFMD patients, 118 (39.3%) were positive for EV71, 61 (20.3%) were positive for CA16, and 26 (8.7%) were positive for other enteroviruses. There was no significant difference in the constituent ratio of enterovirus between adult and child HFMD patients (p = 0.93). The results of RT-PCR were further confirmed by virus isolation assay on human rhabdomyosarcoma cells (RD cells).

**Figure 1:**
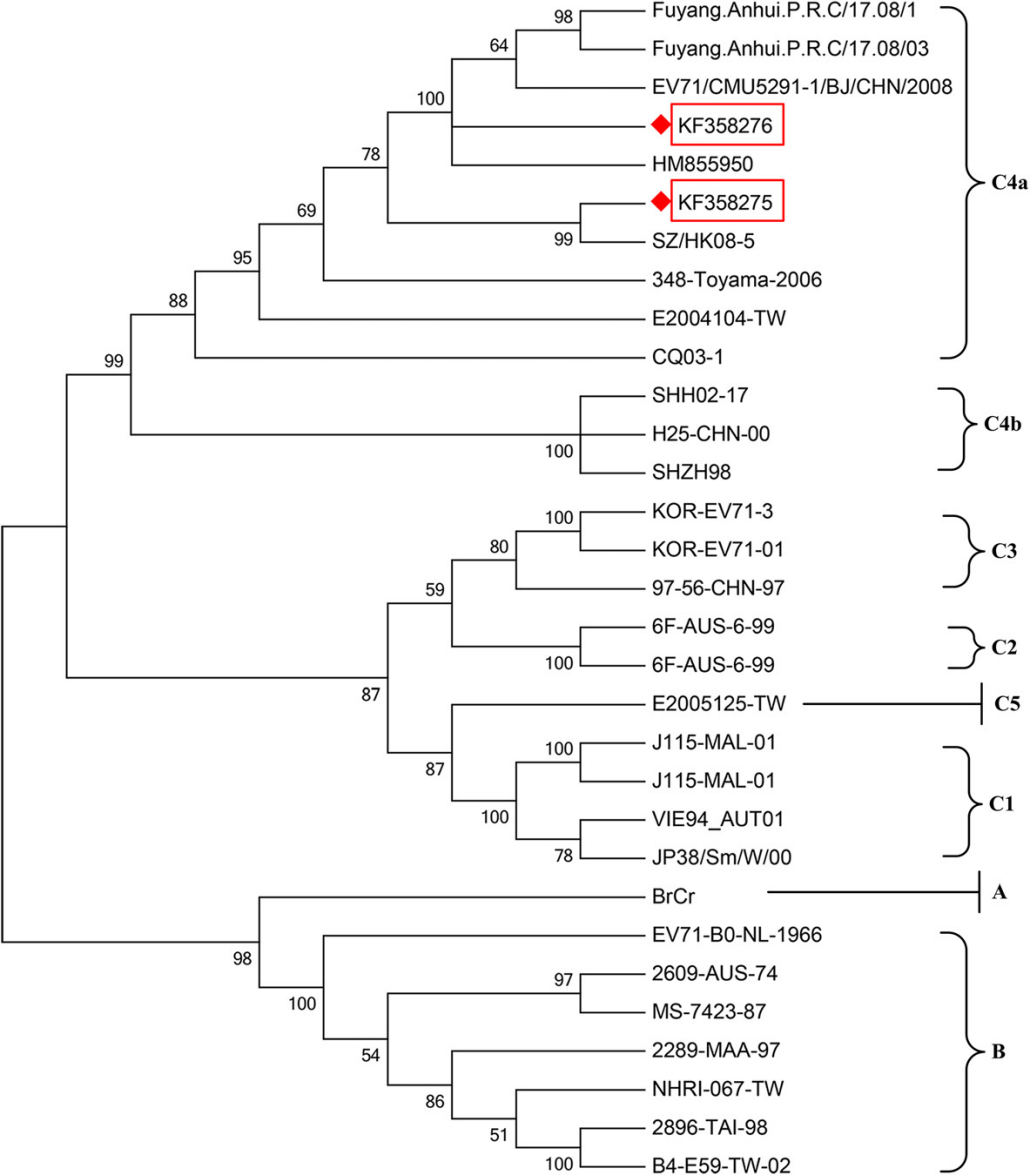
**Phylogenetic analysis of EV71 viruses.** Sequences from adult HFMD patients are highlighted by red rectangles. The newly identified EV71 sequences belong to subgenotype C4a.

Two new sequences encoding the EV71 VP1 were found in samples from adult HFMD patients; these were submitted to the GenBank nucleotide sequence database (accession numbers were KF358275 and KF358276). Phylogenetic analysis showed that these sequences belonged to EV71 subgenotype C4a (Figure 1).

**Figure 2:**
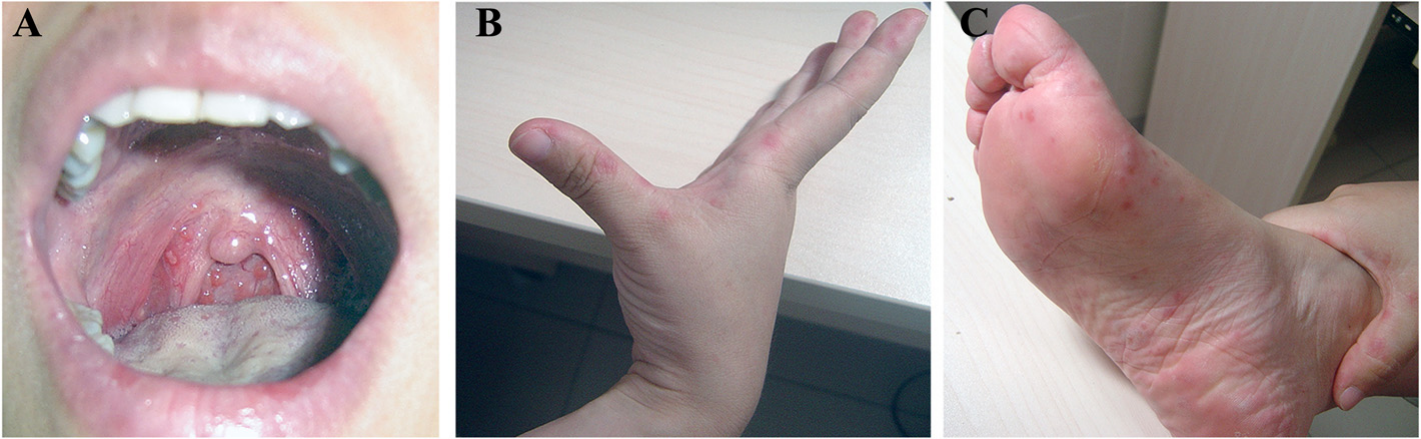
A typical adult HFMD patient presenting with fever, sore throat, and blister-like rash on the (A) oral mucosa, (B) hands, and (C) feet.

A typical adult HFMD patient with herpetic lesions on the hands, feet, and oral mucosa is shown in Figure 2.

### Epidemiological features of adult HFMD

To determine factors associated with adult HFMD, single-factor analysis (α = 0.1) was applied on epidemiological characteristics of adult HFMD patients, with non-HFMD adult patients in the fever clinic serving as controls. Family size ≥4 (odd ratio (OR): 4.27, 95% confidence interval (CI): 1.42–12.83), per capita living space <10 square meters per person (OR: 3.26, 95% CI: 0.96–11.07), having children under 5 years of age (OR: 12.81, 95% CI: 4.81–34.10), having children diagnosed with HFMD (OR: 30.4, 95% CI: 3.87–238.99), and sharing a mobile phone with others (OR: 6.13, 95% CI: 1.63–23.01) were risk factors of adult HFMD (Table 2).

Results of single-factor analysis were further investigated by unconditional logistic regression analysis (likelihood ratio test). Logistic regression analysis showed that having children under 5 years of age (OR: 9.23, 95% CI: 3.18–29.66), having children diagnosed with HFMD (OR: 15.33, 95% CI: 2.12–327.08), and sharing a mobile phone with others (OR: 5.87, 95% CI: 1.25–34.22) were risk factors of adult HFMD (Table 3).

**Table 3:** Risk factors for adult HFMD (logistic regression analysis)

**Epidemiological characters**	**β**	**p value**	**OR (95% CI)**
Family size ≥4 persons	0.47	0.64	-
Per capita living space < 10 square meter/person	0.82	0.41	-
Having children under 5-years-old	2.22	<0.01	9.23 (3.18 ~ 29.66)
Having children diagnosed as HFMD	2.73	0.02	15.33 (2.12 ~ 327.08)
Sharing mobile with others	1.77	0.03	5.87 (1.25 ~ 34.22)

### Demographic features of adult HFMD patients

The geographical distribution and educational attainment of adult HFMD patients was investigated in this study. Of the 33 adult HFMD patients who lived in urban areas, 16 had graduated from college, whereas only two in 16 who lived in rural areas received a college education. Within the 49 non-HFMD adult patients in the fever clinic at the same period, 60% who lived in urban areas had graduated from college, while 15.8% of rural patients received a college education. There was no significant difference in the geographical distribution of educational attainment between adult HFMD patients and non-HFMD adult patients in the fever clinic (p = 0.95).

This study found that 12 female adult HFMD patients were teachers or medical staff members, while only one male adult HFMD patient was a doctor. Of the 49 non-HFMD adult patients in the fever clinic at the same period, six female patients were teacher or medical staff members and three male patients engaged in the same occupations. The job distribution by gender was significantly different between adult HFMD patients and non-HFMD adult patients of the fever clinic (p < 0.01).

### Differences in epidemiological characteristics between adult and child HFMD patients

**Figure 3:**
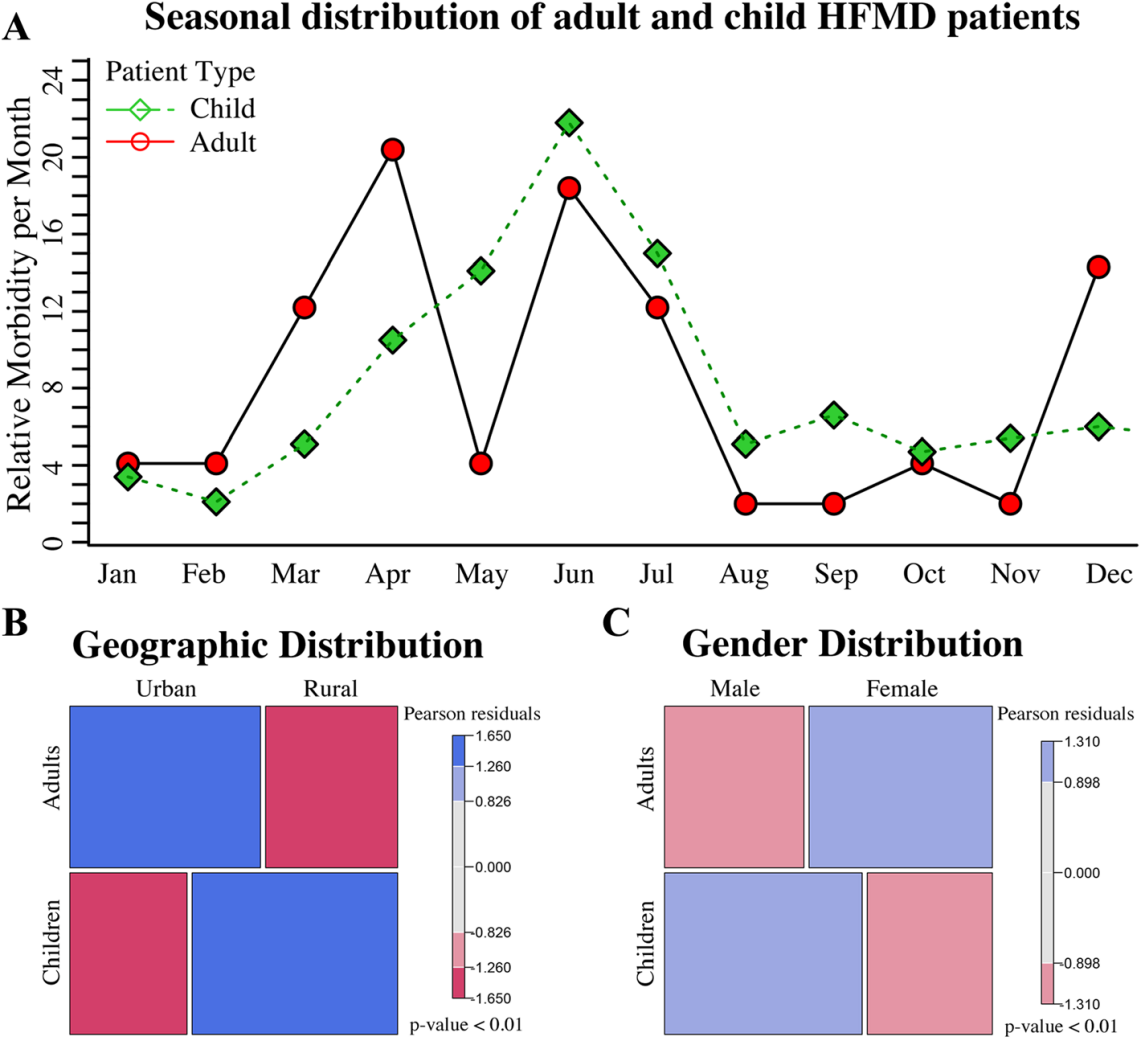
**HFMD epidemiological characteristics of adults compared with those of children. (A)** The peak of HFMD incidence in children was from May to July, while there are peaks in adult HFMD incidence in April, June, and December. **(B)** Children with HFMD were mainly located in rural areas, whereas most of the adult HFDM patients were from urban areas. **(C)** Boys were susceptible to HFMD, while most of the adult HFMD patients were women.

By comparing the case data of 49 adult HFMD patients with those of 8,354 child HFMD patients, several distinct epidemiological patterns of adult HFMD emerged. The peak of HFMD incidence in children was from May to July (Figure 3A, Table 3), which might relate to the survival ability of enteroviruses. In contrast, several peaks in adult HFMD incidence, in April, June, and December, were observed (Figure 3A, Table 4).

**Table 4:** Seasonal distribution of adult and child HFMD patients

**Month**		**Jan**	**Feb**	**Mar**	**Apr**	**May**	**Jun**	**Jul**	**Aug**	**Sep**	**Oct**	**Nov**	**Dec**
Adults n = 49	Number	2	2	6	10	2	9	6	1	1	2	1	7
	Month/Year (%)	4.1	4.1	12.2	20.4	4.1	18.4	12.2	2.0	2.0	4.1	2.0	14.3
Children n = 8354	Number	285	176	425	880	1180	1823	1255	430	551	393	451	505
	Month/Year (%)	3.4	2.1	5.1	10.5	14.1	21.8	15.0	5.1	6.6	4.7	5.4	6.0

Of the 49 adult HFMD patients, 67.3% lived in urban areas, while only 3,032 (36.3%) child HFMD patients resided in urban areas (Figure 3B, Table 5). This difference in geographic distribution was significant (p < 0.01). Additionally, 29 (59.2%) adult HFMD patients were female, whereas only 3,241 (38.8%) child HFMD patients were female (Figure 3C, Table 5), which was also significant (p < 0.01).

**Table 5:** Geographic and gender distribution of HFMD patients: adults vs. children

**Characteristics**	**Gender**				**Geographic**			
	**Male**	**Female**	**χ**^**2**^	**p-value**	**Urban**	**Rural**	**χ**^**2**^	**p-value**
Adults (n = 49)	20	29	8.52	0.004	33	16	20.27	6.71e-06
Children (n = 8354)	5113	3241			3032	5322		

## Discussion

Children and infants are generally susceptible to HFMD, but this disease is uncommon in adults [9,10]. It is speculated that excessive stress, fatigue, and close contact with HFMD patients might contribute to this disease in adults, but the pathogenic mechanism of adult HFMD remains to be well elucidated. To the best of our knowledge, this is the first study to focus on the clinical and epidemiological features of adult HFMD, and is supported by considerable case data collected over 5 years.

All adult HFMD patients in this study had herpetic lesions on the hands, feet, or oral mucosa. However, not all of them had fever, and none had severe complications. Few patients had white blood cell counts over 10,000/μL, and none had X-ray-confirmed pneumonia. In addition, no severe cases were found in this study, and the prognosis of adult HFMD was generally good. These findings suggest that adult HFMD is usually asymptomatic or mild, which is consistent with a prior seroepidemiologic study that about half of the adult population in northern Taiwan had antibodies against EV71 [11]. Thus, adult HFMD patients might ignore the infection and not visit the hospital, which makes them potential reservoirs for human-to-human transmission. Furthermore, physicians might confuse HFMD with viral herpes; a throat swab or anal swab for enterovirus culture would help in making a definitive diagnosis of HFMD.

This study revealed that EV71 was the most common pathogen of adult HFMD, with 10 (31.3%) in 32 specimens positive for EV71. The sequences reported in this study belonged to EV71 subgenotype C4a, which is reported to be the predominant strain in China [12]. The newly identified sequences suggest that EV71 is undergoing continued evolutionary changes. Prior studies reported that EV71 might cause severe neurologic diseases or significant fatalities, and most severe HFMD cases are EV71 positive [13,14]. These findings remind us of the potential threats of EV71 infection among adults.

We show that having children under 5 years of age and having children diagnosed as HFMD were risk factors for adult HFMD. A seroepidemiologic study in Taiwan report that a large proportion of children were EV71 subclinical carriers and served as a reservoir for EV71 spread [15]. It is noteworthy that the median age of adult HFMD patients in this study was 27.5 years and none had underlying disease; these factors could have contributed to there being no severe complications in adult HFMD patients. Taken together, these findings indicate that close contact with HFMD patients or subclinical carriers can lead to a higher risk of exposure to enterovirus.

This study indicated that sharing mobile devices was a risk factor for adult HFMD. Most of the adult HFMD patients played games or watched videos on mobile devices. Many of them shared mobile devices with friends or family members, and some played games or watched videos for more than 1 h per day. However, this study observed that none of the adult HFMD patients routinely sterilized their mobile devices. Mobile devices can be potential reservoirs of viruses [16], which was consistent with the observation in this study that adult patients did not routinely sterilize their mobile devices.

Our study showed that the time distribution of adult HFMD incidence was different from that of child HFMD patients. Prior studies have indicated that meteorological variables such as temperature, humidity, and rainfall are significantly associated with HFMD infection among children [17]. Enteroviruses are reported to survive longest at 20°C and 80% as the humidity [18,19]. May to July is the rainy season in northern Zhejiang with an average temperature at 20°C, which can explain the peak in child HFMD incidence. However, several peaks in adult HFMD incidence were observed in this study, including April, June, and December. Because of the limited number of cases, the decline in the curve in May and the peak in December in adult HFMD incidence cannot be fully explained in this current study.

This study also found a difference in gender distribution between adult and child HFMD patients, which might be related to the fact that women typically have more frequent contact with children than men in the home and at work. In this study, 71.4% of the adult HFMD patients had children under 5 years of age, and 38.8% had children diagnosed with HFMD recently. Because family childcare is typically provided by women, the risk of exposure to enteroviruses is likely higher for women than for men. Additionally, women’s jobs increase the chance for close contact with children. In this study, 41.4% of the female adult HFMD patients were teachers or medical staff members, which have more opportunities for close contact with children than other occupations. However, only 5% of male adult HFMD patients were teachers or medical staff members.

## Conclusion

Close contact with HFMD patients and poor personal hygiene consciousness were risk factors for adult HFMD. Additionally, this study revealed that EV71 genotype C4a was the most common pathogen associated with adult HFMD in northern Zhejiang. A serum epidemiological investigation in Taiwan reported that about half the adult population in northern Taiwan had antibodies against EV71, which implied that EV71 is a highly contagious virus with a high level of transmission [11]. Given that adult HFMD is typically asymptomatic or mild, it was speculated that a large number of adults might be subclinical carriers of HFMD and act as potential enterovirus reservoirs. A seroepidemiologic study in Shanghai documented that the seroprevalence of neutralizing antibodies against EV71 among children was low (only 19.9% were seropositive), which suggested children are generally susceptible to EV71 [20]. Because adults have more social activities than children, it is imperative to prevent the spread of EV71 from adults to children. Moreover, with increasing global travel we should be mindful of the threats of viral transmission by adult HFMD patients with mild symptoms or subclinical disease.

All patients in this study were immunocompetent, and the study did not include HFMD cases who were immunodeficient, such as patients receiving cancer chemotherapy, recipients of an organ transplant, and HIV patients. Therefore, further studies are required to examine the prognosis of HFMD among immunodeficient patients.

## Abbreviations

OR: Odds ratio; CI: Confidence interval; HFMD: Hand, foot, and mouth disease; EV71: Enterovirus 71; CA16: Coxsackievirus A16.

## Competing interests

The authors declare that they have no competing interests.

## Authors’ contributions

YLH and YXG participated in the design, data analysis, and interpretation, and drafted the manuscript. YHX and SJ participated in the design and helped to finalize the manuscript. WXJ and WXJ collected the case data and helped to finalize the manuscript. All authors have read and approved the contents of the final manuscript.

## Pre-publication history

The pre-publication history for this paper can be accessed here:

http://www.biomedcentral.com/1471-2334/14/251/prepub
